# A Scoping Review of Concussion Guidelines in Amateur Sports in the United Kingdom

**DOI:** 10.3390/ijerph19031072

**Published:** 2022-01-19

**Authors:** Emer Scullion, Neil Heron

**Affiliations:** 1 School of Medicine, Dentistry and Biomedical Sciences, Queen’s University Belfast, 97 Lisburn Road, Belfast BT9 7BL, UK; 2 General Practice/Centre for Public Health, Queen’s University Belfast, Belfast BT9 7BL, UK; N.Heron@qub.ac.uk

**Keywords:** SRC, sports related concussion, guidelines, amateur sports

## Abstract

**Objectives** To investigate which United Kingdom (UK) amateur sporting organisations have published sports-related concussion (SRC) guidelines, their accessibility and the extent to which they follow the Berlin statement recommendations. This article is targeted at those involved with designing and implementing SRC guidelines in amateur sport. **Design** Scoping Review. **Data Sources** The SRC guidelines of 15 sporting organisations were accessed through public materials available from the official organisation website. **Eligibility Criteria:** To be included in this review, sports must enjoy broad participation by UK amateur athletes with a high risk of athletes sustaining an SRC. **Results:** 15 sporting organisations were included in this review with two, British Cycling and British Eventing, found not to have published SRC guidelines. There was found to be a large discrepancy between the extent to which the sport-specific guides followed the Berlin statement recommendations. **Conclusions:** The large discrepancy between the contents of the SRC guidelines may be putting the health of athletes at risk. We recommend the UK government publish standardised concussion guidelines based on the latest scientific research that must be used by all UK amateur sport groups.

**What** is already known?

- A recent report by the Digital, Culture, Media and Sports Committee criticised the UK Government and sporting governing bodies for their failure to reduce the risk of SRC, particularly within the amateur athlete population.
- The Berlin statement published by the Concussion in Sport Group (CISG) should act as a framework for the development of SRC guidelines.
- Whilst previous studies have looked at concussion guidelines within elite and professional sport, no research has been conducted focusing on SRC guidelines in amateur sport in the UK.

**What** are the new findings?

- The vast majority of UK amateur sporting organisations have published SRC guidelines following the recommendations outlined by the Berlin statement; however, there is a large discrepancy between the contents of each sport-specific guide.
- We recommend that the UK government publish standardised concussion guidelines for the use of all amateur sporting organisations within the UK.

## 1. Introduction

It is widely recognised in sports medicine that sports-related concussion (SRC) is one of the most difficult injuries to detect, with more needed to be done to recognise, remove and treat athletes with the condition [1]. In July 2021, the Digital, Culture, Media and Sport Committee (DCIS) released a report on concussion in sport ordered by the House of Commons in the United Kingdom (UK) [2]. This report reignited the media’s interest in SRC due to the heavy criticism of the UK Government and sport’s governing bodies for their failure to reduce the risk of SRC, especially within the amateur athlete population.

An SRC is defined as a traumatic brain injury induced by biomechanical forces, either by a direct blow to the head, face, neck or elsewhere on the body with an impulsive force transmitted to the head [3]. Thus, athletes do not need to have a head injury to be concussed, which is a common misconception. The Concussion in Sports Group (CISG) is an organisation formed in 2001 following the first International Conference on Concussion in Sport, with the most recent meeting occurring in Berlin in 2016. Following this meeting, an updated consensus statement was released, building upon the principles outlined in previous statements [4,5,6,7]. The Berlin statement aims to guide those involved with athlete care by providing the latest scientific research on SRC and information on the recognition, diagnosis and management of SRC [8]. Although the statement is not designed to be a clinical practise guideline or legal standard of care, it acts as a framework for the development of sport- specific concussion guidelines published by the governing bodies of sport.

As outlined in the DCIS concussion in sport report, more attention needs to be payed to SRC in the amateur athlete population. Indeed, most sport, in terms of participation numbers, is played at the amateur level. Some aspects of amateur sport may serve to increase the risk of participants suffering from an SRC, such as the reduced access to appropriate protective equipment and the lack of healthcare professionals (HCP) at sporting events and the lack of SRC education for players and coaches. Additionally, some amateur athletes play a number of sports, and it is therefore important that the different SRC guidelines for amateur sports are consistent. This ensures that athletes are not competing in some sports when they are ‘banned’ from playing in other sports due to the different concussion guidelines.

The aim of this scoping review is to investigate the current concussion guidelines used in 15 of the most popular amateur sports in the UK. Similar studies have been conducted focusing on concussion guidelines in elite sport [9] and on elite and amateur sports in the Republic of Ireland [10]. However, this is the first report focusing on amateur sport in the UK. This review will evaluate which sports have concussion guidelines, their accessibility by the general public, and how these compare to the recommendations from the Berlin statement [4,5,6,7], focusing on the methods used to recognise, diagnose and manage athletes with SRC. The main similarities and differences between the sport- specific guides will be discussed, with suggestions made for the improvement of future amateur SRC guides

## 2. Methods

This scoping review was based on the six step methodological framework outlined by Arksey and O’Malley [11] informed by Levac et al. [12]. The Preferred Reporting Items for Systemic Review and Meta- Analysis (PRISMA) was followed with the PRISMA extension for scoping reviews (PRISMA-ScR) checklist [13].

### 2.1. Stage 1: Identify the Research Question(s)

The following research questions were addressed:

1. Which sporting governing bodies have published concussion guidelines appropriate for use in amateur sport in the UK?
2. Do these concussion guidelines follow the recommendations outlined in the Berlin statement?
3. What improvements can be made to future concussion guidelines?

### 2.2. Stage 2: Identifying Relevant Studies

To be included in this review, the sports had to enjoy wide participation at an amateur level within the UK, and participants had to have a high risk of sustaining an SRC by participating in them. Participation levels were taken from the Sport England website [14].

### 2.3. Stage 3: Study Selection

The concussion guidelines for the sporting organisations under review were accessed through public materials published on the official organisation websites. In cases where the concussion guidelines were not available on the website, a relevant organisation representative was contacted to verify the presence or absence of concussion guidelines. Accessibility will be assessed based on the presence of concussion guidelines on these relevant websites, aiming to reflect how an amateur athlete and those involved in their care would search for these guides. When separate organisations were present for the same sport in England, Scotland, Northern Ireland and Wales, the England guidelines were reviewed, as these would typically be used by the largest population of amateur athletes.

### 2.4. Stage 4 and 5: Charting the Data and Collating, Summarizing, and Reporting Results

Microsoft Excel was used to record the relevant data from the articles identified in the literature search. The following data was extracted from the studies: author, year, title, study design, study population and results related to concussion guidelines in amateur sport.

## 3. Results

### 3.1. Sporting Organisation Guidelines

In total, fifteen sporting organisations met the inclusion criteria, with the organisations investigated shown in Table 1. Concussion guidelines were found for all sporting organisations except British Cycling (BC) and British Equestrian (BE), and as a result were excluded from the report.

### 3.2. Compliance with the Berlin Statement

All 13 of the sporting organisations based their concussion protocols on the Berlin statement to varying degrees. Following the convention adopted by the CISG, the findings will be presented using the 11 “R’s” enumerated in the Berlin statement. These include recognise, remove, re-evaluate, rest, refer, recover and return to sport. Rehabilitation, residual effects and risk reduction were not included in this review, as these are more relevant for the use of HCP. Table 2 and Table 3 summarise the contents of the sport-specific concussion guidelines.

## 4. Discussion

### 4.1. Accessibility

In conducting this review, it was found that in most cases the concussion guidelines were not easily accessible on the organisation’s website and required a time-consuming search or required the reviewers to contact an organisation’s representative for clarification. It is not enough for sporting organisations to just publish concussion guidelines, they also need to make these guides readily accessible to the public. Contact had to be made with BG, BE and BC, with no concussion guidelines found for BC and BE, with BG only adding concussion guidelines to their site after researchers drew their attention to their lack of SRC resources. Concussion guidelines have been published for the use of professional athletes in cycling; however, these are not applicable for use at the amateur level, and therefore they cannot be included in this report [29]. Concussion is referenced in the British Equestrian rule book, but was limited to a brief sentence on the role of medical professionals and was not considered enough to constitute concussion guidelines. The delay in access to or lack of concussion guidelines in these sports may be putting participants with an SRC at risk, as it can lead to a delay in diagnosis or the implementation of an incorrect management plan.

### 4.2. Recognise, Remove and Re-Evaluate

It is recognised that in the published literature there is confusion and wide variation in the definition of SRC [3]. Since 2001, the CISG has provided a consistent definition of SRC, which was adopted by all of the sporting organisations in their concussion guidelines. This definition should be used, as it emphasises important SRC factors, such as, for example, that signs and symptoms may only develop hours after the injury, and that concussion is not always associated with a loss of consciousness [8]. Additionally, all of the guidelines provide a comprehensive list of the potential signs and symptoms to look for in athletes who have a potential SRC which aids athletes, their teammates and coaches in recognising the condition. It is worth noting that these signs and symptoms are non-specific to concussion, and therefore guidelines need to recommend further evaluation methods to diagnose SRC.

At present, there is no perfect diagnostic test or marker that clinicians can rely on for an immediate and accurate diagnosis of SRC from the sidelines. As a result, the CISG developed neuropsychological tests that assess cognitive and memory function in athletes with a suspected SRC. The Sports Concussion Assessment Tool 5 (SCAT5) was developed for the use of HCPs and represents the most well-established and rigorously developed instrument available for sideline assessment [30]. It combines the assessment of cognitive symptoms, cognitive status and neurological functioning with separate tools available for adults and children aged 5–12 [31]. The CISG recognised that healthcare professionals are not always present at sporting events, particularly at the amateur level. This led to the development of the Pocket Concussion Recognition Tool (PCRT), which is used as a guide in the recognition of signs and symptoms of possible concussion in ways that can be understood by a layperson [32]. The guidelines published by BB and the BAFA still recommend the use of the SCAT3, which is now the outdated version of the tool and therefore indicates that their guidelines need to be updated. The methods used to assess athletes with a suspected SRC was not mentioned by BJ or EB. These sporting organisations require the presence of medical professionals at competitive events, which may explain the reason for the lack of guidance on this matter. However, concussion can still occur during training sessions without the presence of a trained HCP, therefore they still need to provide athletes with this information. All of the sporting organisations emphasised the importance of removing an athlete with suspected SRC from play immediately for further assessment using the tools discussed, with the athlete not returning to play on the same day as the injury.

### 4.3. Rest

The CISG recommends an initial period of cognitive and physical rest for concussed athletes until they become symptom free. Prescribed rest is the most widely used intervention method for SRC, with the CISG recommending rest for 24–48 h, as followed by all of the organisations except for BB and EN. The reason for this rest period is due to the fact that it may ease symptoms in the acute recovery period by reducing brain energy demands, which may also promote faster recovery [8]. The exact amount and duration of rest has yet to be adequately defined in the literature, with too much or too little rest being detrimental to recovery [33]. Athletes should also be aware that the rest period should involve ‘active rest’, meaning they should gradually increase their physical and mental exertion as long as it does not worsen symptoms. It is important that concussion guidelines include this information to ensure athletes are not prolonging their recovery and subsequent return to sport.

### 4.4. Refer and Recover

While the majority of athletes with SRC recover uneventfully when managed with a brief period of rest and gradual return to play, some may experience persistent symptoms. Persistent symptoms are defined as a clinical recovery that falls outside the expected time frame, >10–14 days in adults and >four weeks in children [34]. Due to the high prevalence of persistent symptoms, which is thought to occur in 10–30% of SRC cases, it is important that sporting organisations provide advice for athletes to follow to help reduce potential morbidity [6,35]. The CISG recommends that anyone with symptoms that fall outside of the normal ranges for their age group must undergo a detailed clinical assessment by an experienced HCP, as followed in the guidelines by BB, EB, ECB, GAA, BG, GBH, EN and ERU [8]. An issue seen in the sport-specific concussion guidelines is the variation in the time frame each organisation uses to define persistent symptoms, with EN and BG the only organisations following the CISG recommendations. Variations include a timeframe of >10 days for BB and EN, >14 days for all players in the GAA, and no timeframe outlined by ECB, GBH and ERU. It is vital that guidelines make athletes aware of persistent symptoms and the importance of seeking medical advice in order to aid recovery and prevent athletes from returning to play before their SRC has fully resolved.

### 4.5. Return to Sport and Reconsider

Premature return to sport is a common issue in amateur sport, with research suggesting 43% of athletes feel they return to play too soon [36]. Sporting organisations should provide athletes with a gradual return to play (GRTP) protocol to ensure athletes return to sport only when they are fully recovered. A GRTP should commence after an initial period of rest (24–48 h) and follow a stepwise approach, with each stage lasting a minimum of 24 h [8]. A GRTP protocol was included in the concussion guidelines of all of the organisations under review, with all of them following the CISG template. The minimum days recommended before return to full contact ranged from six to 35, with GBH and EIHA recommending the shortest recovery period, and EB recommending the longest. While it is recognised that individual athletes will recover at a different pace, the CISG recommends that the GRTP protocol be a minimum of seven days, meaning the GBH and EIHA guidelines are too short and may be putting their athletes at risk. The ECG, GAA and EN all base their recommendations at the lower end of the scale, with minimum return to play occurring seven days after the SRC event.

Boxing has the strictest return to play protocol compared to any of the other sports, with concussed athletes only allowed to return to play after 35 days. Stricter suspension periods are outlined for those who suffer a loss of consciousness (LOC), with the longer the period of LOC equating to a longer suspension period. A LOC for less than one minute is a 90-day suspension with a LOC for greater than one-minute leading to a 180-day suspension. While it is important to implement stricter suspension periods for those who suffer a more serious SRC, this may cause athletes and coaches to only associate a serious concussion with LOC when in fact 90% of concussions occur without an LOC [37].

### 4.6. Paediatric SRC

It is a frequent area of debate as to whether different populations should undergo different RTP protocols, with children being one such example [38]. Concussion in the paediatric and adolescent age group accounts for the majority of SRC due to the large number participating in youth or school sport, with concussion representing an estimated 8.9% of all high school athletic injuries [39]. The EIHA was the only sporting organisation to not include a separate concussion protocol for children. Return to play ranged between 15 days for the GAA to 39 for EB. The rest of the organisations allowed return to play after periods ranging from 23–28 days. The minimum time to return to play was always longer for children compared to adults, with RTP being four–17 days longer for children. All of the organisations with child-specific GRTP followed the recommendations from the CISG, including that child-specific GRTP should refer to children aged 18 and under, and that a brief period of physical and cognitive rest before commencing the GRTP is required, which is then followed by symptom-limited resumption of activity [32]. The more conservative management plan for this age group reflects the evidence in the literature which demonstrates a longer recovery of full cognitive function in younger athletes compared to adults and those playing professionally [40,41,42].

### 4.7. Gender and SRC

The GAA is the only organisation to have separate GRTP protocols for its male and female athletes, with males able to return to the sport at seven days compared to the 14 days required for females. This is based on evidence that female athletes may be at a greater risk of SRC and may take longer to recover than their male counterparts [43,44]. Potential explanations include gender differences in hormones and biomechanics, although it may also be explained by the fact that women have been found to be more honest in reporting injuries than males, potentially explaining the higher SRC incidence levels in this population [45]. More research into gender differences in SRC is required to determine if separate concussion protocols for male and female athletes should be a standard across all sporting organisations.

### 4.8. Return to School/Work

The CISG recommends that all schools should have an SRC policy that should offer appropriate academic support to students recovering from SRC [8]. While the majority of children will be able to return to school with minimal support, some may experience an exacerbation of symptoms or inability to return to school [46]. References to return to work/school were made by all of the sporting organisations except for BB, BG, EIHA and BJ. Information provided by the organisations included making workplaces/schools aware of the athlete’s condition, making any necessary accommodations, and suggesting that the commencement of a GRTP protocol for the sport should occur after a full return to school/work.

### 4.9. Residual Effects and Sequelae

The serious short- and long-term neurological consequences associated with SRC has become an area of increased scientific research, particularly with regard to the effects of sustaining multiple concussions. Multiple concussions have been associated with prolonged symptoms, increased recovery times and leave athletes at a greater risk of sustaining future concussions [47,48,49]. Awareness of the impact of multiple concussions is also important in preventing second impact syndrome, a devastating brain injury that occurs when a second SRC occurs before a previous SRC has fully healed [50]. Sporting organisations should make athletes and their coaches aware of the potential serious consequences of multiple concussions and the appropriate steps to take if they occur. No information on multiple concussions was provided by BAFA, GAA, BG, EIHA, BJ and ERL, meaning these organisations could be putting their athletes at risk. Information included by other organisations was limited to recommending assessment by a trained HCP if two or more concussions occurred within a 12-month period. The most comprehensive multiple concussion guide was provided by EB, which reflects the high risk of multiple concussions in this sport.

The use of a system to record concussions could be a solution to help reduce the number of multiple concussions and the number of athletes returning to sport too early. The sporting organisations that currently have a system to record concussions include BB, EB, ECB, BJ and ERL. Recording concussions means that those involved in athlete care have an accurate record of when and how many SRCs have occurred. The development of a sports-wide concussion reporting system to accurately record all concussion across all sporting organisations may help protect athletes further. At the amateur level, many athletes often play more than one sport and play in different environments e.g., school and clubs, which may increase the risk of multiple concussions if no communication occurs between organisations. The development of this reporting system would mean that all those involved in the athlete’s care would have access to an accurate concussion history and can treat the individual accordingly.

### 4.10. Future Recommendations

This scoping review has emphasised the large discrepancy between the contents of the concussion guidelines published by UK sporting organisations. As a result, we recommend the development of a single UK-wide concussion protocol that is based on the latest scientific evidence. We also recommend that this protocol must be readily accessible to amateur athletes and all those involved in their care. The development of a standardised SRC protocol was also one of the main recommendations made by the UK Government in their recent ‘Concussion in Sport’ review. The report stated that within the next 12 months this standardised protocol should be published and “used by National Governing Bodies as the minimum standard in creating the rules for their sport” [2]. Currently, Scotland is the only country within the UK to have a standardised SRC protocol, which should act as an example for England, Wales and Northern Ireland [51]. It is not enough for the UK government to just publish SRC guidelines; they also must deliver a comprehensive communication campaign to ensure that everyone involved in sport, from the athletes, parents, coaches and HCPs can improve SRC outcomes. The campaign needs to emphasise the importance of recognising and managing the condition correctly and include details about where people can access the information so they can take action in the event of an SRC.

### 4.11. Limitations

Limitations of this review include the fact that we only researched the guidelines of 15 of the most widely participated amateur sports in the UK. We recognise that there are other amateur sports in which athletes may sustain a SRC which have not been included in this scoping review. We also recognise that this report is limited to UK sporting organisations, with further research needing to be done before the recommendations from this report can be applied to SRC guidelines in other countries.

## 5. Conclusions

Whilst the health benefits of physical activity far outweigh the potential risks of serious injury, it is important that athletes have an awareness of what to do if an injury such as an SRC occurs. The development of accessible sport-specific SRC guidelines is vital in providing athletes and those involved in their care with the correct tools to recognise, diagnose and manage SRC. This report found that that the vast majority of UK sporting organisations have published concussion guidelines following the recommendations outlined by the CISG, but that there is a large discrepancy in the contents of the sport-specific guides. This could be putting the health of athletes at risk, particularly at the amateur level, where concussion protocols are often implemented without the advice of a trained HCP. We recommend that the government should publish standardised SRC guidelines that should be followed by all UK amateur sporting organisations in order to provide the latest evidence-based SRC diagnostic and management practices.

## Figures and Tables

**Table 1:** British sporting organisations under review.

Sport	Sporting Organisation Concussion Guidelines	Sport(s) Played within Organisation
American Football	British American Football Associatio [15]	American Football
Basketball	British Basketball [16]	Basketball and Wheelchair Basketball
Boxing	England Boxing [17]	Boxing
Cricket	England and Wales Cricket Board [18]	Cricket
Cycling	British Cycling	Road, Off road (MTB), Track, Paracycling, Amateur BMX, Leisure/Sportive & Cyclo-cross
Gaelic	Gaelic Athletic Association [19]	Gaelic, Hurling, Camogie and Handball
Gymnastics	British Gymnastics [20]	Acrobatic Gymnastics, Aerobic Gymnastics, Disability Gymnastics, Men’s Artistic Gymnastics, Rhythmic Gymnastics, TeamGym, Trampoline, Double Mini Tramp, Tumbling and Women’s Artistic Gymnastics
Hockey	GB and England Hockey [21]	Field Hockey
Equestrian	British Eventing [22]	Dressage, show jumping and cross country
Ice Hockey	England Ice Hockey Association [23]	Ice Hockey
Judo	British Judo [24]	Judo
Netball	England Netball [25]	Netball
Rugby League	England Rugby League [26]	Rugby League
Rugby Union	England Rugby Union [27]	Rugby Union, Sevens Rugby, Tag and Touch Rugby
Soccer	Football Association [28]	Soccer/Football

Abbreviations: BAFA, British American Football Association; BB, British Basketball; EB, England Boxing; ECB, England Cricket Board; BC, British Cycling; GAA, Gaelic Athletic Association; BG, British Gymnastics; GBH, Great British Hockey; BE, British Eventing; EIHA, England Ice Hockey Association, BJ, British Judo; EN, England Netball; ERL, England Rugby League; ERU, England Rugby Union; FA, Football Association.

**Table 2:** Summary of SRC guidelines published by UK sporting organisations.

Sporting Organisation	Assessment Tools	Initial Complete Rest (Hours)		GRTP Protocol	Return to Play		Notes
		Child	Adult		Child	Adult	
BAFA	SCAT3 PCRT	48	24	Yes	23	19	Athletes should not be left alone, consume alcohol or drive until all symptoms have gone
BB	SCAT3 PCRT	NA	NA	Yes	23	19	NA
EB	NA	24–48	24–48	Yes	39	35	Do not stay alone for the first 24 h post- injury Minimise use of mobile phones, TV, reading and all forms of training and exercise
ECB	SCAT5 PCRT	NA	24	Yes	23	7	No alcohol, prescription or non- prescription drugs
GAA	SCAT5 VOMS CRT5	48	Male- 24–48 Female- 48	Yes	15	Male- 7 Female- 15	Should not be left alone for first 24 h. Minimise exposure to TV, PC, laptops, smartphones, tablets, video games etc
BG	SCAT5 PCRT	NA	48	Yes	28	14	NA
GBH	SCAT5 PCRT	NA	24	Yes	23	6	Must be off all medications that modify symptoms of the concussion e.g., painkillers
EIHA	SCAT5 PCRT	NA	24–48	Yes	NA	6	NA
BJ	NA	7–10 days	7–10 days	Yes	28	14	If unconsciousness results from shime waza (strangulation technique) the player may be allowed to return following three days of rest
EN	SCAT5 PCRT	NA	NA	Yes	23	6	If no doctor present is suitably trained and experienced in the management of SRC a mandatory two week rest period must occur before commencing GRTP for all ages
ERL	SCAT5 PCRT	48	24	Yes	23	19	Individuals should avoid initially then gradually reintroduce; reading, TV, computer games and driving
ERU	SCAT5 PCRT	24–48	24–48	Yes	23	19	No driving, exercise, minimise screen time No cognitive (brain) activities e.g., reading, television, computer, video games and smart phones.
FA	SCAT5 PCRT	24–48	24–48	Yes	23	19	Should not be left alone within the first 24 h, consume alcohol or drive a motor vehicle

Abbreviations: SCAT—Sports Concussion Assessment Tool; PCRT—Pocket concussion recognition tool; VOMS—Vestibular Ocular Motor Screening; GRTP—Gradual return to play.

**Table 3:** Summary of SRC guidelines published by UK sporting organisations continued.

Sporting Organisation	Prolonged Recovery	Return to Work/Education	Record Concussion	Multiple Concussions	Medical Clearance
BAFA	NA	A player may need a day or two off work/study to rest and reasonable adjustments made to the player’s normal work/study	NA	NA	No
BB	Concussion lasting longer than 10 days needs specialist assessment	NA	NA	A second concussion within 12 months should be assessed and managed by HCPs	Yes
EB	If symptoms >four weeks post injury for children or >two weeks for adults contact your GP	When going back to school, you may need to go back gradually and have some changes to your schedule so that symptoms do not worsen. You should not return to sport until you have returned to full school/learning without symptoms	Yes	Two knockouts in 90 days = 90- day suspension Three knockouts in 12 months = 1-year suspension Loss of Consciousness (LOC): <1 min = 90- day suspension >1 min = 180- day suspension Three LOC within a 12-month period = 18-month suspension	Yes
ECB	Urgent neurological or neurosurgical consultancy before continuing GRTP	Return-to-school guidelines, which include extra-time for assignments/exams, quiet study areas, increased breaks and rest	Yes	Player needs a prolonged recovery period (i.e., three weeks) and/or onward referral.	Yes
GAA	Symptoms lasting > 10–14 days should be referred to an appropriate specialist	A graduated return to school/education strategy is necessary.	NA	NA	Yes
BG	Individuals with symptoms >4 weeks for children and >2 weeks for adults may benefit from a supervised multidisciplinary approach	NA	NA	NA	Yes
GBH	Should be assessed and managed by experienced HCP	Athletes should partake in a graduated return to school programme.	NA	Should be assessed and managed by an experienced HCP	Yes
EIHA	NA	NA	NA		Yes
BJ	NA	NA	Yes	NA	Yes
EN	Recovery >10 days need referred to an experienced HCP	Academic and non- academic work should cease until stage two of the GRTP and that workload is reduced until completion of GRTP	NA	If second concussion within a 12-month period or history of multiple concussions (>3) referral to experienced HCP should take place	Yes
ERL	NA	It is reasonable for a student to miss a day or two of studies but extended absence is uncommon and the young person’s academic teacher(s) or tutor should be consulted	Yes	NA	Yes
ERU	Athletes who fail to progress through GRTP should return to their GP for review	In some cases, it may be appropriate for the player to miss a day or two of work/study	NA	Anyone who sustains >2 concussions in a 12-month period should seek advice from their GP	No
FA	NA	Students may need to have allowances made for impaired cognition during recovery e.g., extra time	NA	Any player with a second concussion within 12 months should be assessed and managed by an experienced HCP	Yes

Abbreviations: HCP- Health care professional; GRTP- gradual return to play.

## Data Availability

All data generated or analysed during this study are included in this published article.
